# The bidirectional relationship between weight, height and dental caries among preschool children in China

**DOI:** 10.1371/journal.pone.0216227

**Published:** 2019-04-30

**Authors:** Anqi Shen, Eduardo Bernabé, Wael Sabbah

**Affiliations:** Faculty of Dentistry, Oral & Craniofacial Sciences, King’s College London, London, United Kingdom; Centre Hospitalier Regional Universitaire de Tours, FRANCE

## Abstract

There is evidence of a bidirectional association between dental caries and anthropometric measures among children. This dual relationship has not been examined in the same population. The objectives of this study are (1) to examine the relationship between baseline caries and changes in weight and height; and (2) to assess whether baseline weight and height are associated with changes in dental caries in a sample of preschool Chinese children. Children were recruited from 15 kindergarten in Liaoning Province at baseline (8 in rural area and 7 in urban area), a total of 1,111 of children were included at baseline. The mean age of children at baseline was 50.82 months. Data were collected through clinical oral examination, assessment of anthropometric measures and structured questionnaire. Dental caries was assessed according World Health Organization (WHO) methods by one dentist. Sociodemographic and behaviour data were also collected. At follow-up, 772 children were included (attrition rate: 30%), dental caries and anthropometric measures were assessed again. Z-score for weight-for-age and height-for-age were calculated using the 2006 and 2007 WHO Child Growth Standards. The sum of decayed, missing and filled primary teeth (dmft) were used in the analysis. Multilevel analysis for longitudinal data was conducted to explore the relationship between z-score for weight-for-age and height-for-age, and dental caries among children. The median follow-up time was 10.12 months. There was a significant negative association between dmft at baseline and change in height-for-age. On the other hand, weight-for-age at baseline was negatively associated with change in dmft at follow-up. The findings suggest that dental caries impedes children’s growth indicated by height for age. Low weight children appear to be more susceptible to dental caries in the same population.

## Introduction

Dental caries is one of the most prevalent diseases in China affecting more than half of children and majority of adults [1]. With 97% of decayed teeth among 5-year-olds left untreated, the disease undoubtedly has implications on children’s wellbeing [2]. Undernutrition is another common and persisting problem in China. In 2011, approximately 19% of 2–6 years old children were underweight and 4% were stunted [3]. Although the rate of undernutrition in children is gradually declining, China is still facing a huge challenge of nutrition imbalance among children. On the other hand, the shifts in diet and activity led to an increase in children’s and adults’ obesity [4]. The prevalence of obesity increased to 10.1% in 2010, and then remained stable until 2014 among 5–6 years children [5]. Moreover, the rapidly changing lifestyles are highly associated with changes in disease patterns. The unhealthy lifestyles include excessive consumption of sugar and high fat diet [6].

Studies have argued that dental caries is correlated with increasing weight, which was attributed to sugar consumption [7–11]. Other studies argued that dental caries negatively impacts children’s growth, practically in low income countries and communities [12–16]. Direct effects of dental caries, associated pain and inflammation impact children’s ability to eat and result in poor dietary intake that contributes to stagnation of weight and height gain [17]. On the other hand, there is evidence that being malnourished, underweight or stunting might affect the development of dental cavities [18–20]. No known study has examined this bidirectional relationship in the same population of preschool children. Furthermore, the economic and nutritional transitions in China, and the patterns of obesity, undernutrition and dental caries occurring simultaneously among Chinese children provide a good opportunity for assessing this bidirectional association. Identifying this bidirectional association would be helpful in informing health promotion policies aiming at tackling problems associated with children’s anthropometric measures and dental caries among children. Furthermore, the study could help integrate interventions aiming at reducing the burden of dental caries, child obesity and undernutrition.

This longitudinal study examined the association between weight and height-for-age and dental caries among preschool Chinese children. The objectives of this study are to assess whether there is an association between dental caries at baseline and change in weight and height, and whether there is an association between weight and height at baseline and changes in dental caries.

## Materials and methods

Ethical approval for this study was obtained from King’s College London (KCL Ethics Ref: HR-15/16-2901). An oral consent was also given by Shenyang Dental Hospital (Ministry of Health of People’s Republic of China). A written consent was obtained from participants’ parents prior to baseline and follow-up data collection. The following formula was used to calculate the sample size: N = (z_α_ + z_β_)^2^ / (δ/σ) ^2^ (z_α_ = significance, z_β_ = power, δ = difference between the baseline and follow-up means, σ = pooled standard deviation of means) [21, 22]. The sample size calculation is based on previous studies of the association between dental caries and anthropometric measurements among preschool Chinese children. Based on a study that examined the relationship between weight and height, and dental caries (outcome) among preschool children in Taiwan [23], the calculated sample size with 0.05 significance level and 80% statistical power was 278 and 176 for weight and height, respectively. Based on a study estimating weight and height (outcomes) among children with and without dental caries in Hong Kong [24], the sample size was 636 (significant 0.05, power 80%). The highest sample size of these three estimates was rounded up to 650. This number was increased to a minimum of 1000 to compensate for non-response and attrition at follow-up.

### Study population

**Fig 1:**
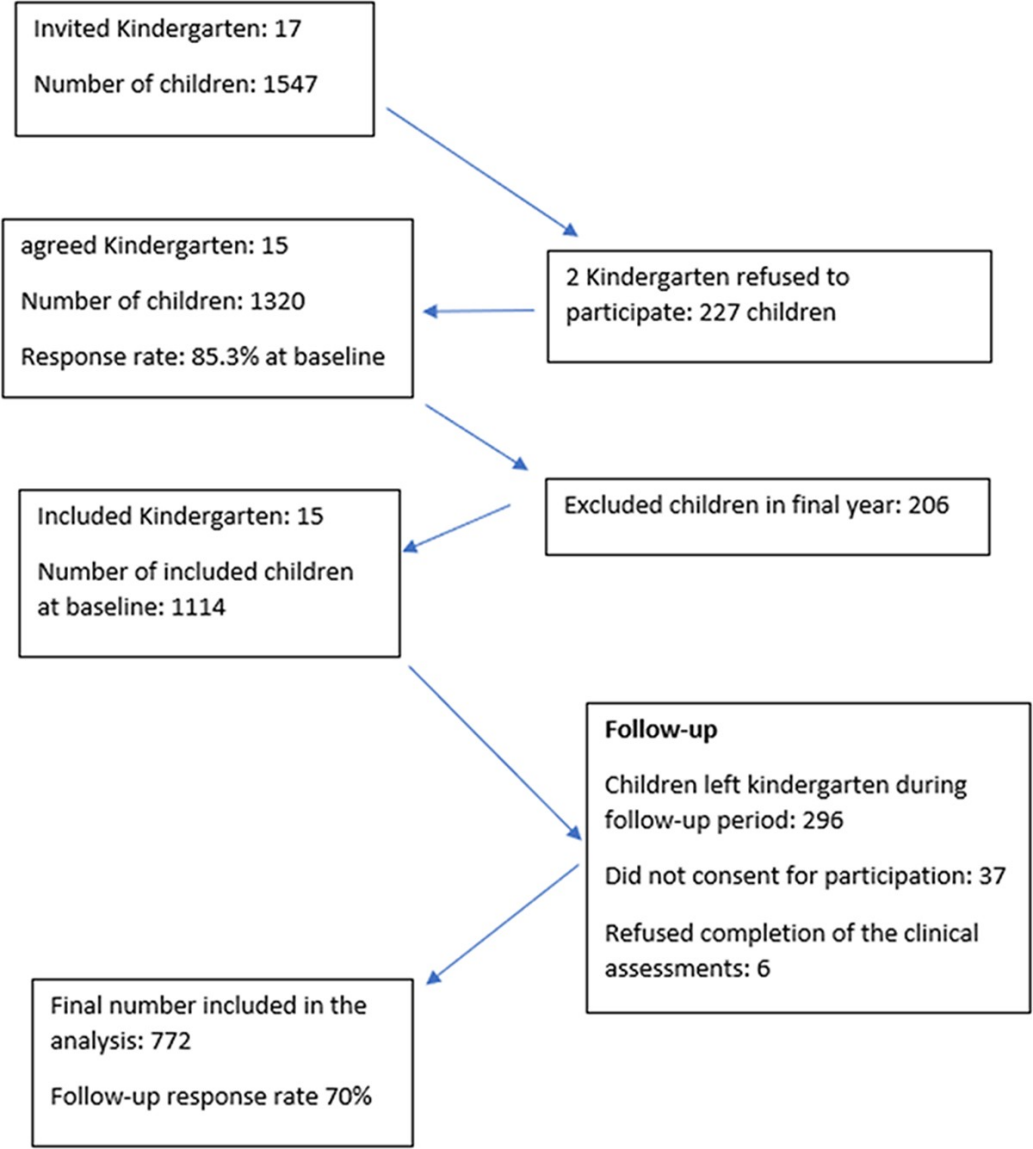
Flowchart of participation at baseline and follow-up.

Data was collected from preschool children living in Shenyang, Liaoyang and Fushun, Liaoning province, China. The Shenyang dental hospital hosts an annual screening programme for assessing dental caries. This study was incorporated with the hospital screening programme. While the hospital staff collected information on presence or absence of caries, for the purpose of this study, dental examination according to WHO survey methods was conducted. Baseline data was conducted from October 2016 to early January 2017. Follow-up data was collected on July, October and November 2017. At baseline, 17 kindergartens with 1547 participants were approached to be included in the study. However, 15 kindergartens were included at baseline due to refusal to participate by two kindergartens. Eight kindergarten were in rural area and seven in urban area. Children in the 15 kindergartens were all selected into this study. The number of potential participants dropped to 1320 with response rate of 85.3% at baseline. Children in final years were excluded as they would not be around at follow-up bringing the sample to 1114 children. Three children refused to complete clinical assessment reducing sample size to 1111 at baseline. At follow-up, 30% of the included sample at baseline were lost either because they left the kindergarten (296 children), did not consent for participation (37 children) or refused completion of the clinical assessments (6 children). A total of 772 children at follow-up were finally included in the analysis (Fig 1). The mean age of children at baseline and follow-up was 50.82 and 60.55 months, respectively. The mean and median follow-up time was 9.73 months (SD: 1.22) and 10.12 months, respectively.

### Exclusion criteria

1. Children who did not provide a consent form from their parents at baseline or follow-up

2. Children in final year at baseline

### Data collection

Data was collected through clinical oral examination and assessment of anthropometric measures. Structured questionnaire was modified from that used in the Third National Oral Health survey in China [2]. The questionnaire was disseminated to participants’ parents to collect data on socioeconomic and demographic factors, dietary habits, oral and general health of the children. Data on socioeconomic (income) and demographic factors (age, gender and area) were extracted from questionnaires. Income was categorised into five categories: 0–3999, 4000–5999, 6000–9999, 10000 Chinese yuan and undeclared. Feeding pattern in the first six months of age was categorised into exclusively or mainly breastfeeding versus others. Frequency of visiting a dentist in the past 12 months was categorized into three groups: once or more, no visit and do not recall. Toothbrushing was categorised into less than once a day/other versus once or more. Parents’ reported children’s general health was classified into excellent or good versus poor or others. Hospitalisation (admission to hospital) within the past 12 months was classified into Yes or No. This variable was included for potential impact on children’s growth. Fresh fruits and sugar consumption were assessed as continuous variables. Sugar consumption was calculated based on overall intake frequency of four snacks and drinks (biscuits, cake or bread; candy or chocolate; sweetened water and soft drinks; and fresh fruit). Each snack or drinks, and fresh fruit were assigned a score as follows: twice or more a day (2), once a day (1), 3–4 times a week (3/7 days = 0.43), once a week (1/7days = 0.14), once a month (1/30 days = 0.03), and seldom or never (0) [25]. Weighted scores of four snacks and drinks were then summed to produce an overall score. The score was used to indicate daily frequency of intake of sugars for each participant. Fresh fruit consumption was used as a separate variable.

Only one dentist conducted dental examination and recorded information in the oral examination forms. The dentist attended training prior to data collection on using WHO standard criteria for assessing dental caries [26]. The Intra-examiner reliability was assessed among 80 participants in two different days (baseline and follow-up). The level of agreement (Kappa) was 0.72, which is considered substantial agreement. Participants were asked to sit in a chair and an artificial light source was used in oral examination. All participants were asked to brush their teeth at home in the morning prior to oral examination. Dental status in primary dentition was assessed by dmft index, which was used to calculate caries experience. Anthropometric measures were assessed by one person working in Shenyang dental hospital using anthropometric standard methods. The person who did anthropometric measurements was blinded to children’s dental status. Participants were asked to wear light clothing and remove their shoes. The weight and height of participants were measured with a weight and height scale to the nearest 0.5 kilograms and 0.5 cm. The equipment was recalibrated prior to the procedure.

Age- and sex-specific percentile for weight/height were used to determine the weight/height status of the children rather than actual weight/height used for adults [27]. Children’s weight/height were classified using thresholds that vary to consider the children’s age and gender. These thresholds are usually derived from a reference population, known as a child growth reference. They are calculated by weighing and measuring a large sample of children and they illustrate how weight/height varies in children of different ages and sex. As well as showing the pattern of growth, these data also provide an average weight/height for a boy or girl at a particular age, and the distribution of measures above and below this value. This means that individual children can be compared to the reference population and the degree of variation from the expected value can be calculated. Z-score is widely recognized as the best system for analysis and presentation of anthropometric data for children because of its advantages compared to the other methods [28]. The WHO Growth Standards were used to convert weight and height measures to z-scores, namely weight-for-age and height-for-age [29, 30]. The WHO Growth Standards 2006 was applied to children aged 24–60 months and the WHO Growth Standards 2007 for children aged more than 60 months [31].

### Statistical analysis

All the participants at follow-up (772 children) were included to conduct descriptive and multilevel analysis. All analysis was conducted using STATA 15.0 [32]. First, we assessed the distribution of socioeconomic (income) and demographic factors (age, gender and area), oral health behaviours (feeding patterns, frequency of visiting dentist and frequency of toothbrushing), parents-reported general health and hospitalisation of the children in the past 12 months at baseline. Second, the description of anthropometric measurement (actual weight and height, and z-score for age-for-weight and height) and dmft index at baseline and follow-up were also assessed.

Multilevel linear regression models were used to assess the study objectives. Participants’ age in years was used as time variable in all models and fitted as a continuous time indicator. The time interval between baseline and follow-up was adjusted for in all models. Both intercept and slope with time were considered as random effects to examine changes in weight and height for age, and dmft over time. No constrains were imposed on the values of the covariance matrix (unstructured model).

We first examined change in weight-for-age z-score within the follow-up period, then baseline dmft was added to the fixed effect model. Finally, a fully adjusted model was constructed (adjusting for age, sex, income, parents’ reported children’s general health, hospitalisation, fresh fruit and sugar consumption). The same sets of models were constructed with height-for-age z-score as the outcome.

Multilevel analysis was also used to explore the relationship between weight-for-age/height-for-age z-scores at baseline and change in dmft. Model 1 assessed change in dmft during follow-up period, and time was applied into both fixed and random effects. In the second model, the association between change dmft and baseline z-score for weight was examined. Finally, a fully adjusted model was constructed including all variables associated with dmft (age, sex, income, feeding patterns in the first six months of age, frequency of visiting dentist, frequency of toothbrushing, fresh fruit and sugar consumption). The same sets of models were used to assess the association between baseline height-for-age z-score and change in dmft over time.

## Results

Among those who were lost at follow-up, mean age was 51.15 months, percentage of boys and girls were 51.10% and 48.94%, mean dmft was 3.17, means of actual weight and height were 18.56 and 106.26, respectively. There were minimal differences between those who were lost at follow-up and those completed the study in these parameters.

In Table 1, mean age at baseline and follow-up was 50.82 and 60.55 months. The percentages of boys and girls were 51.42% and 48.58%, respectively. Children living in rural area were 65.54% of total population. The highest percentage of income groups are second lowest (20.98%) and second highest (20.98%), followed by lowest (16.71%) and highest (13.99) income groups. Overall, 49.09% of children were exclusively or mainly breast fed in the first six months, and 36.79% children cleaned their teeth once or more a day. Only 14.25% of the children visited a dentist once or more in the last 12 months. Parents reported children’s general health as excellent or good for 52.33%, and 11.66% children were admitted to hospital over the past 12 months.

**Table 1:** Description of all variables at baseline (n = 722).

Variables	N	%	(95%CI)
Mean age at baseline	50.82		(50.09, 51.55)
Mean age at follow-up	60.55		(59.84, 61.26)
Gender			
Male	397	51.42	(47.89, 54.95)
Female	375	48.58	(45.05, 52.11)
Area			
Urban	266	34.46	(31.18, 37.89)
Rural	506	65.54	(62.11, 68.82)
Income			
0–3999	129	16.71	(14.25, 19.52)
4000–5999	162	20.98	(18.25, 24.01)
6000–9999	162	20.98	(18.25, 24.01)
10000 or above	108	13.99	(11.71, 16.63)
Undeclared	211	27.34	(24.30, 31.59)
Feeding patterns			
Exclusively or mainly breastfeeding	393	49.09	(45.57, 52.63)
others	379	50.91	(47.37, 54.43)
Dental visits past 12 months			
Once or more	110	14.25	(11.95, 16.90)
None	415	53.76	(50.22, 57.26)
Don’t recall/ others	247	31.99	(28.79, 35.38)
Frequency of toothbrushing			
Less than once a day or others	488	63.21	(59.74, 66.55)
Once or more	284	36.79	(33.45, 40.26)
Parents’ reported children’s general health			
Excellent or good	404	52.33	(48.79, 55.85)
Poor or Others	368	47.67	(44.15, 51.21)
Hospitalisation past 12 months			
Yes	90	11.66	(9.56, 14.13)
No	682	88.34	(85.87, 90.43)
Mean Fresh fruit consumption	1.41		(1.37, 1.45)
Mean Sugar consumption	1.03		(0.97, 1.09)

In Table 2, the mean weight for age z-score was 0.58 at baseline and increased to 0.66 at follow-up. The mean height for age z-score increased from 0.49 at baseline to 0.69 at follow-up. The mean dmft index was 3.18 at baseline and 4.21 at follow-up.

**Table 2:** Description of anthropometric measures and dental caries at baseline and follow-up.

	Baseline		Follow-up	
	Mean	95%CI	Mean	95%CI
dmft index	3.18	(2.91, 3.45)	4.21	(3.90, 4.51)
Weight-for-age z-score	0.58	(0.50, 0.66)	0.66	(0.58, 0.75)
Height-for-age z-score	0.49	(0.42, 0.56)	0.69	(0.62, 0.76)
Weight	18.36	(18.07, 18.64)	20.58	(20.24, 20.91)
Height	106.47	(105.93, 107.01)	112.91	(112.38, 113.44)

Table 3 shows factors associated with change in weight- and height-for-age z-scores. There were significant increases in weight and height-for-age overtime with regression coefficient 0.09 (95% CI: 0.05, 0.13) and 0.21 (95% CI: 0.18, 0.24), respectively. There was no significant association between baseline dmft and change in weight-for-age (Coefficient: -0.02; 95% CI: -0.04, 0.01). Dental caries at baseline (dmft) was negatively and significantly associated with change in height-for-age z-score (Coefficient: -0.03; 95% CI: -0.04, 0.01) in the unadjusted model. The model adjusted for all factors also showed significant association between dmft index and height-for-age (Coefficient: -0.02; 95% CI: -0.04, 0.01).

**Table 3:** Multilevel linear analysis of factors associated with changes in height and weight for age over one year.

	Weight for age			Height for age		
	Model 1	Model 2	Model 3	Model 4	Model 5	Model 6
	Coefficient (95% CI)	Coefficient (95% CI)	Coefficient (95% CI)	Coefficient (95% CI)	Coefficient (95% CI)	Coefficient (95% CI)
dmft index		-0.02 (-0.04, 0.01)	-0.02 (-0.04, 0.01)		-0.03** (-0.04, -0.01)	-0.02** (-0.04, -0.01)
Age (year)	0.09*** (0.05, 0.13)	0.10*** (0.06, 0.13)	0.10*** (0.06, 0.14)	0.21*** (0.18, 0.24)	0.22*** (0.19, 0.25)	0.22*** (0.19, 0.25)
Income (ref: 0–3999)						
4000–5999			0.36** (0.10,0.61)			0.18 (-0.05, 0.41)
6000–9999			0.23 (-0.02, 0.48)			0.22 (-0.01, 0.45)
10000 or above			0.26 (-0.03, 0.54)			0.20 (-0.06, 0.46)
Undeclared			0.19 (-0.05, 0.44)			0.06 (-0.16, 0.28)
Parents’ reported children’s general health (ref: poor/other)						
excellent or good			-0.01 (-0.17, 0.15)			-0.01 (-0.15, 0.14)
Hospitalisation past 12 months (ref: No)						
Yes			-0.09 (-0.33, 0.14)			-0.08 (-0.30, 0.14)
Fresh fruit consumption			-0.04 (-0.19, 0.12)			-0.01 (-0.14, 0.14)
Sugar consumption			-0.01 (-0.10, 0.08)			0.01 (-0.07, 0.09)

*** p<0.001

** p<0.01

In Table 4, there was positive increase in dmft overtime (Coefficient: 1.29; 95% CI: 1.16, 1.41), and negative association between weight-for-age at baseline and change in dmft (Coefficient: -0.24; 95% CI: -0.46, -0.03). The association remained significant after adjustment for potential confounders (Coefficient: -0.22; 95% CI: -0.43, -0.01). Children who were exclusively breastfed (Coefficient: 1.20; 95% CI: 0.71, 1.69) and children with more sugar consumption (Coefficient: 0.32; 95% CI: 0.06, 0.58) had greater increase in dmft. Although the direction of this association showed a negative relationship between height-for-age at baseline and change in dmft, but the relationship was not significant.

**Table 4:** Multilevel linear analysis of factors associated with changes in dmft over one year.

	Model 1	Model 2	Model 3	Model 4	Model 5
	Coefficient (95% CI)	Coefficient (95% CI)	Coefficient (95% CI)	Coefficient (95% CI)	Coefficient (95% CI)
Weight-for-age z-score		-0.24* (-0.46, -0.03)	-0.22* (-0.43, -0.01)		
Height-for-age z-score				-0.23 (-0.46, 0.01)	-0.18 (-0.41, 0.05)
Age (year)	1.29*** (1.16, 1.41)	1.29*** (1.17, 1.41)	1.29*** (1.17, 1.41)	1.29*** (1.17, 1.41)	1.29*** (1.16, 1.41)
Income (ref: 0–3999)					
4000–5999			0.30 (-0.47, 1.08)		0.25 (-0.53, 1.02)
6000–9999			-0.40 (-1.16, 0.37)		-0.43 (-1.19, 0.34)
10000 or above			-0.74 (-1.60, 0.12)		-0.77 (-1.64, 0.09)
Undeclared			-0.04 (-0.81, 0.73)		-0.07 (-0.84, 0.69)
Feeding patterns (ref: exclusively/mainly bottle feeding or others)					
Exclusively/mainly breastfeeding			1.20*** (0.71, 1.69)		1.19*** (0.70, 1.68)
Frequency of visiting dentist (ref: once or more)					
0 time			-0.67 (-1.37, 0.03)		-0.67 (-1.37, 0.03)
Don’t recall or others			-0.11 (-0.98, 0.76)		-0.10 (-0.98, 0.77)
Frequency of cleaning teeth (ref: less than once a day or others)					
once or above a day			-0.17 (-0.74, 0.40)		-0.14 (-0.71, 0.42)
Fresh fruit consumption			0.13 (-0.34, 0.60)		0.13 (-0.34, 0.60)
Sugar consumption			0.32* (0.06, 0.58)		0.32* (0.06, 0.58)

*** p<0.001

*p<0.05

## Discussion

The findings of this study on the bidirectional association between anthropometric measures and dental caries indicated by dmft among preschool children in China showed that dental caries at baseline was negatively and significantly associated with children height over the follow-up time. The direction of the association between baseline caries and weight gain was also negative, but it was not statistically significant. On the other hand, children with higher weight-for-age at baseline had lower caries increment.

Other longitudinal research demonstrated an inverse association between dental caries and changes in Body Mass Index (BMI) among children [33]. Severe dental caries might affect ability to eat, disrupt sleep, lead to loss of appetite and accelerate adverse effects of other risk factors in children’s nutritional status through chronic infection or inflammation [34]. Dental caries results in stunting and low BMI that could contribute to an adverse impact on quality of life, including pain, lack of sleep, concentration, hunger and absenteeism form school [35]. Alkarimi et al (2014) argued that untreated severe dental caries might negatively impact height and weight gain through immune, endocrine or metabolic response or directly through limiting eating abilities [17]. The findings of the current study confirm a negative impact of caries on children growth, particularly height gain.

There are several longitudinal studies assessing the association between weight/height and change in dental caries among children. While a lack of association was occasionally reported [36], several studies, mostly in developed countries, demonstrated a positive relationship between obesity and caries increments [7–11], a relationship mostly attributed to eating habits. On the other hand, other studies argued that malnourishment among children would result in increase in caries increment [18, 20, 37], which was in accordance with the results of this longitudinal study. Malnutrition could affect oral tissues and dental cavities development [19]. Deficiencies of vitamin A and D and lack of protein and other micronutrients such as vitamins, zinc and iron appear to limit the protective effect of saliva on the dental cavity [19]. Undernutrition could also cause tooth decay through an impact on enamel formation and also children’s chewing abilities [34]. It is worth noting that the current analysis uniquely demonstrated a bidirectional association between caries and anthropometric measures in the same children population.

Several cross-sectional studies showed different views on the relationship between weight/height and dental caries among Chinese children. Findings from a recent Chinese research were consistent with this longitudinal study showing children with higher weight more likely to have lower dental caries [38]. Peng (2017) concluded that underweight children were more likely to have more caries than normal weight children, and obese children have a lower prevalence of dental caries than normal weight children [38]. Another Chinese research also supported the same view. Liang (2016) demonstrated that children with higher BMI had lower odds of caries with overweight and obese Chinese children more likely to be caries free in primary dentition [39]. Similarly, Yang (2015) found an inverse relationship between BMI and dmft index among Chinese children [40]. However, some research showed conflicting views on this relationship. Yao (2014) argued that higher BMI was associated with higher prevalence of dental cavities among 5–14 year-old Chinese children [41]. Another paper reported that weight/height z-score was associated with prevalence of dental caries experience (dmft>0) (24). Others did not find an association between dental caries and obesity among children in Taiwan (23) and in mainland China [42].

In this study, children who were exclusively breast-fed in the first six months were found higher increments of dental caries in this study. Most research emphasized that children who had been bottle-fed had higher risk of having dental caries compared with children who were breast-fed [43, 44]. However, Perera (2014) revealed that breast-fed children had a higher prevalence of dental caries than bottle-feeding children [45]. Higher prevalence of caries among exclusively breast-fed children might be due to these children had higher prevalence of overnight feeding [45].

The relationship between weight/height and dental caries could also be confounded by other factors. It is worth noting that the bidirectional associations between caries on one hand, and weight and height on the other were independent from a number of risk factors related to the development of dental caries such as sugar consumption, dental visits, oral hygiene and family income, and risk factors related to children growth such as general health, dietary habits and income. Unsurprisingly, sugar consumption was associated with change in dental caries, which is consistent with the literatures on the role of sugars in the development of caries [46, 47]. Obesity, dental caries and other chronic diseases share common risk factors, and reducing the risk of common factors might decrease the risk of chronic diseases [48]. This study provides some implications for future studies. Controlling risk factors such as sugary diet appears to promote general health and decrease the prevalence of many chronic diseases in a more efficient and effective way [48].

There are a few limitations worth mentioning in this study. First, some participants were lost to follow-up (attrition rate: 30%). However, this is inevitable in longitudinal studies, particularly when children are recruited from kindergartens where attendance is not compulsory in China and children could move out for any change in their parents’ circumstances. Furthermore, there were minimal differences in age, gender, dmft index, weight and height at baseline among those who completed the study and those who were lost at follow-up. Second, the use of decayed, missing and filled surfaces index (dmfs) could be viewed as superior to dmft, particularly in studies aiming at assessing population treatment needs. However, for the purpose of the current analysis, the presence of cavitation was deemed adequate. Third, some health-related factors were reported by their parents, which could be subjected to recall bias, but there was no other alternative as access to medical records was not possible. Finally, a longer follow-up period could have allowed better observation of changes in the outcome variables. This however was inevitable due to the short period the children spend at kindergarten.

This study along with results from earlier studies have some implications for public health policies, particularly in countries experiencing the dual burden of obesity and undernutrition among children such as China. Healthcare systems in developing countries prioritise life threatening conditions at the expense of what is seen as less threatening health problems such as dental caries. The potential impact of dental caries on undernutrition observed in this study and other studies in developing countries highlights the need for prioritising oral health [49], not only for the elimination of pain and restoration of functional dentition but also to avoid a negative impact on children’s growth and general wellbeing. That aside, dental caries and obesity share common risk factors and reducing these factors could decrease the risk of both conditions [48]. Dietary intake, particularly that including high sugar, is an established common risk factor for both dental caries and weight gain [50]. Controlling such common risk factors could potentially promote oral and general health and reduce chronic diseases in a more efficient and effective way.

## Conclusions

This is the first longitudinal study to examine the bidirectional associations between dental caries, height and weight among preschool children in China. The study demonstrated a significantly negative relationship between baseline dental caries and children growth indicated by height-for-age, and between baseline weight and caries increment. The findings highlight the importance of dental caries as a potential risk factor for children growth in China and the role of nutritional status in the development of caries.
